# Inferring Behavioral States of Grazing Livestock from High-Frequency Position Data Alone

**DOI:** 10.1371/journal.pone.0114522

**Published:** 2014-12-04

**Authors:** Hermel Homburger, Manuel K. Schneider, Sandra Hilfiker, Andreas Lüscher

**Affiliations:** 1 Agroscope, Institute for Sustainability Sciences, Reckenholzstrasse 191, CH-8046, Zurich, Switzerland; 2 University of Freiburg, Faculty of Biology, Geobotany, Schaenzlestrasse 1, D-79104, Freiburg, Germany; Deakin University, Australia

## Abstract

Studies of animal behavior are crucial to understanding animal-ecosystem interactions, but require substantial efforts in visual observation or sensor measurement. We investigated how classifying behavioral states of grazing livestock using global positioning data alone depends on the classification approach, the preselection of training data, and the number and type of movement metrics. Positions of grazing cows were collected at intervals of 20 seconds in six upland areas in Switzerland along with visual observations of animal behavior for comparison. A total of 87 linear and cumulative distance metrics and 15 turning angle metrics across multiple time steps were used to classify position data into the behavioral states of walking, grazing, and resting. Five random forest classification models, a linear discriminant analysis, a support vector machine, and a state-space model were evaluated. The most accurate classification of the observed behavioral states in an independent validation dataset was 83%, obtained using random forest with all available movement metrics. However, the state-specific accuracy was highly unequal (walking: 36%, grazing: 95%, resting: 58%). Random undersampling led to a prediction accuracy of 77%, with more balanced state-specific accuracies (walking: 68%, grazing: 82%, resting: 68%). The other evaluated machine-learning approaches had lower classification accuracies. The state-space model, based on distance to the preceding position and turning angle, produced a relatively low accuracy of 64%, slightly lower than a random forest model with the same predictor variables. Given the successful classification of behavioral states, our study promotes the more frequent use of global positioning data alone for animal behavior studies under the condition that data is collected at high frequency and complemented by context-specific behavioral observations. Machine-learning algorithms, notably random forest, were found very useful for classification and easy to implement. Moreover, the use of measures across multiple time steps is clearly necessary for a satisfactory classification.

## Introduction

In animal ecology, researchers aim at understanding how individuals or groups interact with their environment. They are faced with the challenge of observing tracks and behavior of the studied animals more or less continuously over extended time periods. Important questions are habitat preferences and resource utilization [1], travel routes and behavior in migratory species [2], and social and predatory interactions within or between species [3]. In pasture ecology, grazing behavior and resting time of domestic grazers are of major interest when studying efficient resource use, productivity, and impacts on ecosystem functioning [4]. Walking, grazing, and resting are the primary activities of grazing livestock and have a large impact on pasture ecosystems [5]. Selective grazing is considered to be the most important impact on pasture vegetation [6]. Grazing can increase or decrease heterogeneity of vegetation, depending on preexisting vegetation patterns and on the strength of plant-soil interactions [7]. Walking and thus trampling of animals leads to soil compaction and is a potential source of soil erosion. Resting is often associated with deposition of excreta which can, together with herbage removal by grazing, lead to a large-scale redistribution of nutrients over the pasture area [8]. By reason of their importance for pasture ecosystems, we are focusing on the mentioned three activities within the current study.

Gathering data about animal behavior holds essential difficulties. Direct observation by humans can be hindered or impossible, e.g. in adverse weather conditions or at night, or if the observations must be carried out continuously over space and time. Furthermore, human presence might alter the behavior of the observed animals. These limitations are more and more overcome nowadays by the use of remote-observation techniques, so-called bio-logging, which have been increasingly explored in the last decade in studying animal movement and behavior [9]. Telemetry, such as the Argos system, has been designed to deliver positions over long time periods, but with limited positional accuracy. It is frequently used with marine animals [10], [11], migratory birds [12] or migrating terrestrial mammals [13]. On the other side of the spectrum are the Global Navigation Satellite Systems such as the Global Positioning System (GPS). Their receivers have a high energy demand and limited battery life but they can deliver positional data with high accuracy and at short time intervals. Bio-logging techniques thus offer great advantages compared to human field observations. But at the same time they require the assignment of obtained positions to different behavioral states of the animals under study. The determination of behavior can be supported by the use of additional sensors, such as accelerometers to measure quick and local movements of the legs, heads or entire bodies of animals [14]–[16], heart rate monitors to measure energy costs of activity [17] or noseband pressure sensors to determine bite rate and rumination behavior [18]. Using GPS receivers alone avoids energy demand and costs of additional sensors and reduces manipulations to the animals' bodies. Therefore, the question arises how to gain behavioral information from position data alone and to which extent this is possible.

The differentiation of behavioral states is especially challenging if movement patterns are predominantly slow and complex. The behavioral activities of pasturing cows, sheep or goats, for example, change frequently within a relatively small space, making their distinction difficult [16]. Several studies have presented approaches to behavioral classification of grazing livestock from position data alone. Schlecht et al. [19] measured positions of Zebus at 10 s resolution and classified positions using four movement metrics within a three minute time interval as predictor variables in a linear discriminant analysis. Anderson et al. [20] tracked beef cows at time intervals of 1 s and used threshold values of mean movement rate calculated for one minute periods to discern between walking, grazing, and resting.

The accuracy of behavioral classification depends on several factors. First, measuring accuracy of the remote tracking device matters [21], [22]. Second, behavioral variation between individuals or breeds of the same species, as well as different animal species, can play a role in the determination of distinct behavioral states [23]. Third, the time interval between GPS measurements in the field can influence classification accuracy [24]. And fourth, the result may depend on the statistical approach used for classification. Quantitative studies dealing with behavioral classification of bio-logging data often apply process modelling using state-space models [25] or machine learning such as classification trees [15] and linear discriminant analysis [19]. Machine learning methods group data points based on some measure of similarity. They are easy to implement and flexible but ignore the temporal and spatial process which underlies the data. State-space models retrace the underlying process by explicitly modelling data in the temporal and spatial order in which they have been recorded. They are also able to integrate additional sources of errors (e.g. device accuracy) [25] but they require more efforts for implementation and higher computational power.

Our aim was to compare several machine learning algorithms and state-space modelling regarding behavioral classification. Temporal dependence was modelled explicitly with the state-space model, and implicitly within the machine learning models by using a range of predictor variables computed over multiple consecutive positions. Specifically, we addressed the following questions:

1. Is it possible to differentiate between the behavioral states of walking, grazing, and resting of cows grazing upland pastures based on GPS positions alone? Quantifying livestock behavior (e.g. walking, grazing, and resting) at low costs and limited manipulations on the animals' bodies greatly facilitates investigating relationships between livestock and the ecosystem at a local scale.

2. What is the effect of model parameterization, temporal resolution of the data, and classification technique on classification accuracy in total and for the individual behavioral states?

3. How is the abundance of the behavioral states influenced by the classification? Such activity budgets are of great interest in the assessment of management impact on grazing livestock in the long term.

## Materials and Methods

### Study sites

The study was conducted on six grazed upland areas, so-called alpine farms, at elevations between 1,400 and 2,400 m asl. The farms are only temporally grazed and inhabited during summer. They were situated in two Swiss mountain regions, the canton of Obwalden in the Northern foothills of the Alps and the Lower Engadine (canton of Grisons) in the Eastern Central Alps. All farms were grazed by dairy cows, two of them additionally had suckler cows. The size of single paddocks varied between 0.17 ha and 87 ha, and herd size varied between 15 and 120 cows. The vegetation was mainly composed of montane and subalpine grassland types, dwarf shrub associations, and some forested pasture areas. Animals under study were in private ownership and handled by the owners. No further permits were required for the described measurements, which complied with all relevant regulations.

### GPS tracking

On each of the alpine farms, three to four cows were equipped with GPS collars. The aim was to register a range of individual behaviors that represented the majority of the cows on a farm. Therefore, cows were selected that showed ordinary behavior and were well integrated into the herd. A leather saddle carrying the logger box was mounted directly on the bell collar of each cow selected for study. The differential GPS loggers were low-cost models (Qstarz BT-Q1000XT, Qstarz Ltd., Taipei, Taiwan) with EGNOS correction [21], a recording capacity of 400,000 waypoints and a maximum logging frequency of 1 Hz. In order to extend the measuring period, the device was modified with two 3.6 V lithium batteries, lasting up to six weeks. Considering that we attempted to collect measurements for the duration of the battery life, we determined that a recording interval of 20 seconds was a reasonable trade-off between data storage capacity and information content. Our decision was reinforced by a preliminary study indicating similar visual interpretability of tracks recorded at 10 s and 20 s time intervals. Measurements were done throughout the entire summer, 2011. After three to six weeks, the stored positions were transferred to a computer and projected from WGS84 (EPSG: 4326) to the Swiss national grid CH1903 LV95 (EPSG: 21781).

With three of the loggers we evaluated the measurement accuracy of the devices. Each was placed on fixed points with known coordinates for up to twenty minutes. The fixed points were located in hilly terrain at locations with both full and limited satellite visibility. We calculated the deviation of the logged positions from the fixed point position (absolute error), as well as the deviation of the logged positions from their centroid (relative error). The 0.5 percentile of the absolute error was at 3.1 m, the 0.95 percentile was at 4.39 m. For the relative error, the 0.5 percentile was at 1.49 m, and the 0.95 percentile was at 2.44 m.

### Visual observation of behavior

In order to obtain training and validation data for the classification, the behavior of fifteen cows, each equipped with a GPS logger, was observed on the pasture for up to six hours each. Single observation sequences of one to three hours were dispersed over the entire period of measurement and the six study areas. Some cows were observed twice, resulting in nineteen track sequences containing visual observations of behavior and predictor variables. The observer followed the herd at a distance ensuring that the cows behaved undisturbed. Animal behavior was observed continuously, i.e. every behavioral change of the cow was recorded together with precise GPS time, based on which behavior was assigned to the GPS positions at discrete time intervals. Observed behaviors were grazing, walking, running, resting, standing, interacting with other animals of the herd, defecating, and drinking. Interacting, defecating, and drinking were too rare for classification, and as we were interested in those behaviors exercising the greatest influences on the ecosystem we only considered the three behavioral states walking, grazing, and resting for further evaluations. Walking was observed when the cow moved more than five steps with the head up, and comprised also running. Resting comprised standing and lying. Grazing was recorded when the cow was ripping the grass off and also when it made a few steps forward with the head down, in search of food. In so doing we defined the behavioral states broadly to reduce very quick changes of observed behavior in our data. The original data set is provided as Dataset S1 under the PLOS ONE data policy.

### Movement metrics

**Figure 1:**
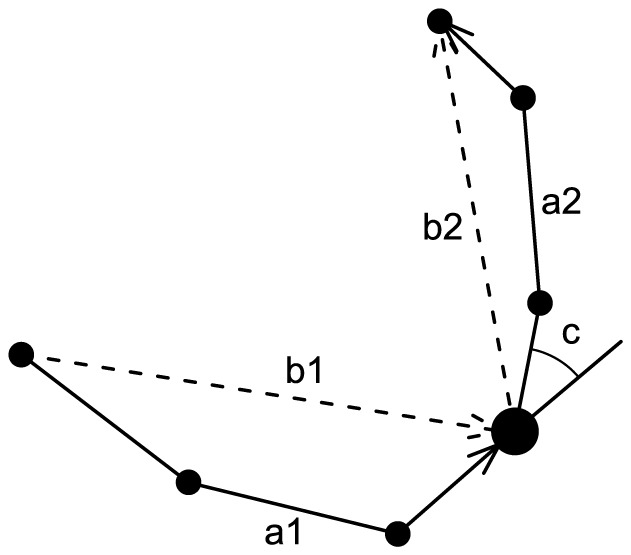
Schematic representation of movement metrics used as predictor variables in the classifications. Movement metrics include backward cumulative distance (a1), forward cumulative distance (a2), backward linear distance (b1), forward linear distance (b2), and turning angle between GPS positions (c).

From position data alone, different movement metrics can be derived and used for the identification of behavioral states. The two basic metrics are the distance between subsequent positions and the turning angle at each position (Figure 1). They are often used in state-space models to discern between behavioral states. In a first step, we compared the density distributions of the two basic metrics between the states walking, grazing, and resting. Since larger tracking time intervals can hinder the differentiation of behavior based on the two described basic movement metrics, we subsampled the original data set at 60 s time intervals and recalculated the metrics.

In a second step, we extended the two movement metrics distance and turning angle over multiple positions. Our motivation was the fact that each of the cows' activities often lasts for several minutes, and so, movement integrated over a longer time period may be meaningful in describing cow activities from position data alone. Regarding distance, we computed two types of metrics, cumulative distances along positions and linear distances between positions (Figure 1), both of which were computed at each 20 s interval, up to five minutes before and after each position. In the following text, distances that lie temporally before a considered position are called backward distances, and those after a focal position are called forward distances. In addition, we computed the average of backward and forward distances for each time interval (20 s, 40 s, 60 s, etc., up to five minutes) and each distance type (cumulative, linear). Regarding turning angle, we generated average turning angles over three, five, seven, etc… positions up to five minutes around each position. The resulting 87 distance metrics and 15 turning angle metrics were added to each position as predictor variables.

### Evaluated models

Eight models were evaluated, which differed in a) the classification approach, b) the preselection of training data, and c) the number and type of predictor variables (Table 1).

**Table 1:** Characterization of the eight classification models.

Model	Classification approach	Preselection of training data	Predictor variables
A	RF	-	1 distance + turning angle
B	RF	-	87 distances + 15 turning angles
C	RF	RUS	87 distances + 15 turning angles
D	RF	SMOTE	87 distances + 15 turning angles
E	RF	60 s interval + RUS	27 distances + 5 turning angles
F	LDA	SMOTE	87 distances + 15 turning angles
G	SVM	SMOTE	87 distances + 15 turning angles
H	SSM	-	1 distance + turning angle

Shown are the classification approach (RF  =  random forest, LDA  =  linear discriminant analysis, SVM  =  support vector machine, SSM  =  state-space model), the method of preselection (RUS  =  random undersampling, SMOTE  =  synthetic minority oversampling technique) and the number and type of predictor variables.

The evaluated classification approaches were a random forest algorithm (models A – E), a linear discriminant analysis (model F), a kernel support vector machine (model G), and a state-space model (model H). The random forest algorithm is a machine learning algorithm especially suited for data sets with many, and possibly highly collinear, predictor variables [26], [27]. We used package randomForest 4.6-7 in R 3.0.2 [28]. Linear discriminant analysis (function lda in R package MASS 7.3-29 [29]) seeks linear combinations of predictor variables to describe the levels of a categorical variable [29], [30]. Kernel-based machine learning methods extract structure from the data by an “implicit mapping of the input data into a high dimensional feature space defined by a kernel function” [31]. We built a support vector machine with Gaussian Radial Basis kernel using R package kernlab 0.9–19 [31]. Model H was a state-space model, in which the sequence of observed behavioral states was modeled as a mixture of three random walks with fixed switching probabilities between them [32]. Each random walk was characterized by a distinct combination of turning angle and distance to the preceding position, which followed a wrapped Cauchy and a Weibull distribution. Model parameters were estimated by Markov Chain Monte Carlo using JAGS 3.4.0 [33]. Wide Gamma distributions were used as vague priors for the Weibull parameters and uniform distributions for the wrapped Cauchy as well as for switching probabilities, respectively. Convergence was rapid and assessed with Gelman-Rubin statistics [34]. In order to limit computing time, posterior samples were drawn from 500 iterations of three chains each, after discarding 200 iterations for burn-in.

In order to avoid classification bias due to uneven class sizes, training data was preselected using two different techniques: random undersampling (models C and E) and synthetic minority oversampling (models D, F and G). No preselection was carried out prior to models A and B, and neither to model H, as in state-space models the whole course of a track is needed for modelling. Random undersampling (RUS) was achieved by drawing a random sample of positions from each of the three behavioral states the size of the smallest class (walking) for each tree of a random forest. RUS is implemented in the R random forest function, specified with “sampsize” and “strata”. In contrast, using the synthetic minority oversampling technique (SMOTE), new artificial data for the least frequent state were added by extrapolating from the original data [35]. It is implemented in the package DMwR [36]. In addition, only positions every 60 s were used for model E, in order to investigate how a lower temporal resolution changed the classification accuracy. For the subsampled data set, movement metrics for up to five minutes before and after each position were recalculated.

Regarding the predictor variables, models A and H were built with only the distance to the preceding position and the turning angle at a position, which is a frequent constellation in random walk models [37]. All 87 distances and 15 turning angles were used in models B, C, D, F, and G. Only 27 distance variables and 5 turning angles were used in model E because of the larger time interval after data thinning. In addition, a null model was built, where behavioral states were randomly assigned to positions in the abundance in which they occurred in the dataset.

### Model testing and evaluation

Similar to real-life situations, where GPS tracks are classified based on relatively short sequences with behavioral observations, classification accuracy was quantified based on the prediction of independent track sequences. In turn, each observed sequence of behavioral states was predicted from models fitted to the remaining 18 sequences. In the state-space approach, the most frequent state in the posterior sample of each position was taken as the predicted state. The percentage of correctly classified positions was calculated for each sequence individually and jointly for all available observations. Differences of classification accuracy between the models were tested for statistical significance with a t-test.

Besides classification accuracy, we were also interested in how well spatial and temporal patterns of animal behavior can be modeled, i.e. which of the positions are predicted correctly and which not. Hence, a short example track representing a small subset of the data was selected. For the example track, predictions of one realization of the eight models at each position were visualized and the confusion matrices were calculated. In order to give an impression of how the model influences the estimation of the animals' behavioral budget, for each of the eight models the relative abundance of behavioral states was determined within the example track sequence as well as within the whole data set.

For better ecological interpretation of the multiple predictor variables we determined their individual importance for the classification of each of the behavioral states. The calculation of variable importance is implemented within the R function randomForest [28]. As an illustrative example, variable importance was evaluated based on model C.

## Results

### Characteristics of observation data set

**Figure 2:**
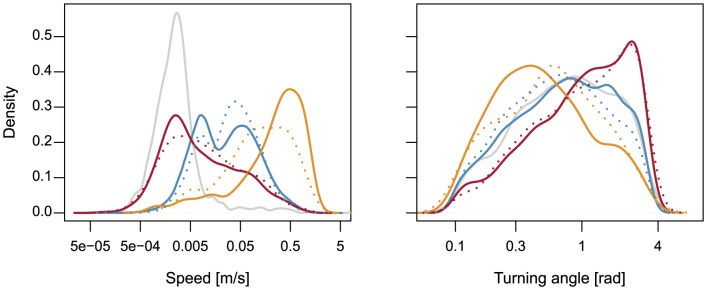
Distributions of movement speed and turning angle at 20 s and 60 s time intervals. Density distributions within the observed behavioral states walking (gold), grazing (blue), and resting (red), and in the data set of the test of GPS device accuracy (gray). Continuous lines represent the original data with 20 s intervals, dotted lines represent the data subsampled to 60 s intervals.

The 19 individual track sequences containing visual observations of behavior covered a total of 44 hours. The data set was unbalanced for the three behavioral states: 478 positions were observed as walking, 6674 positions observed as grazing, and 2296 positions observed as resting. Observed walking, grazing, and resting differed with respect to the movement speed of the cows (Figure 2). The distributions of speed values for the three observed behavioral states showed separate peaks, but there was a considerably large overlapping area between resting and grazing, and a smaller one between grazing and walking. Several positions, where resting was observed, had rather high speed values. The stationary measurements from the test of device accuracy demonstrated a narrower distribution. Speed values between positions observed as grazing formed two distinct peaks. Density distributions of speed values calculated after subsampling the original data to 60 s intervals showed larger overlapping areas, i.e. more similar values between behavioral states than those at 20 s intervals. Remarkably, the distributions for walking and resting in the 60 s dataset were flatter than in the original 20 s data, but the distribution for grazing was steeper and narrower (Figure 2). Mean movement speeds of the cows were (with 0.05 and 0.95 quantiles in brackets) at walking 0.41 m/s (0.005 m/s, 1.08 m/s), at grazing 0.06 m/s (0.003 m/s, 0.25 m/s), and at resting 0.04 m/s (0.0007 m/s, 0.2 m/s). The distributions of turning angle values for walking, grazing, and resting were less separated in the original 20 s data than those of movement speed (Figure 2). In the 60 s data, the turning angle distributions for walking and grazing were almost completely overlapping, whereas the distribution for resting only changed marginally. Interestingly, the distribution of turning angle within the stationary measurements corresponded largely to the one within grazing. Mean turning angles of the cows were (with 0.05 and 0.95 quantiles in brackets) at walking 0.63 rad (0.03 rad, 2.21 rad), at grazing 0.92 rad (0.05 rad, 2.66 rad), and at resting 1.21 rad (0.07 rad, 2.88 rad).

### Classification accuracy of the models

**Figure 3:**
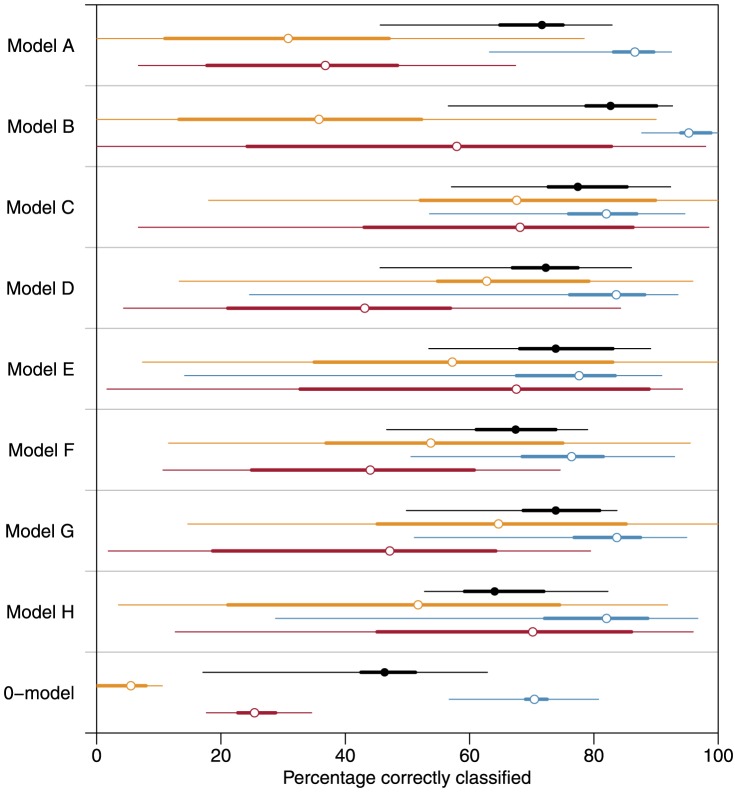
Classification accuracies of the models A – H and the null model. Percentage of correctly classified states in joint sequences together with 50% and 95% quantile intervals for individual sequences, overall (black) and for the three behavioral states walking (gold), grazing (blue) and resting (red). For specifications of models see Table 1.

Model A, based on the unbalanced data set and one distance plus one turning angle as predictor variables, yielded an overall classification accuracy of 72% with a highly unbalanced state-specific accuracy (Figure 3). Adding distance measures and turning angles over multiple time steps improved the classification accuracy significantly (P<0.001), reaching 83% in model B. Using this model, grazing behavior was classified with an accuracy of 95%. However, resting (58%) and walking (36%) were classified less accurately. Balancing the data with RUS (C) led to a non-significant drop of classification accuracy (77%) compared to model B. However, with balanced data, the specific accuracies for the three behaviors were more similar (walking: 68%, grazing: 82%, resting: 68%). Balancing with SMOTE (D) also led to an equalization of the state-specific classification accuracies, but to a smaller extent than RUS, and the overall classification accuracy was somewhat lower (72%) than that of model C and significantly (P<0.01) lower than that of model B. Classification accuracy of model E, based on data thinned to 60 s intervals, was 74%, which is close to the average of all evaluated models. The same overall accuracy was reached with the support vector machine (G), but with more unbalanced state-specific accuracies despite data balancing with SMOTE. The linear discriminant analysis (F) and the state-space model (H) produced the lowest classification accuracies compared to all other models with 67% and 64%, respectively. The overall accuracy of model H was lower than for model A with the same predictor variables. However, state-specific accuracies were more similar in model H than in model A. The null model had an overall classification accuracy of 46% with the expected uneven state-specific accuracies of 5% for walking, 70% for grazing, and 25% for resting.

There was also a remarkably large variability in the classification accuracy for the two states of walking and resting (Figure 3). Most of this variability resulted from the fact that these two states were very rare in some sequences and hence the misclassification of a few positions resulted in a low classification accuracy.

### Classification of individual observations in an example track

**Figure 4:**
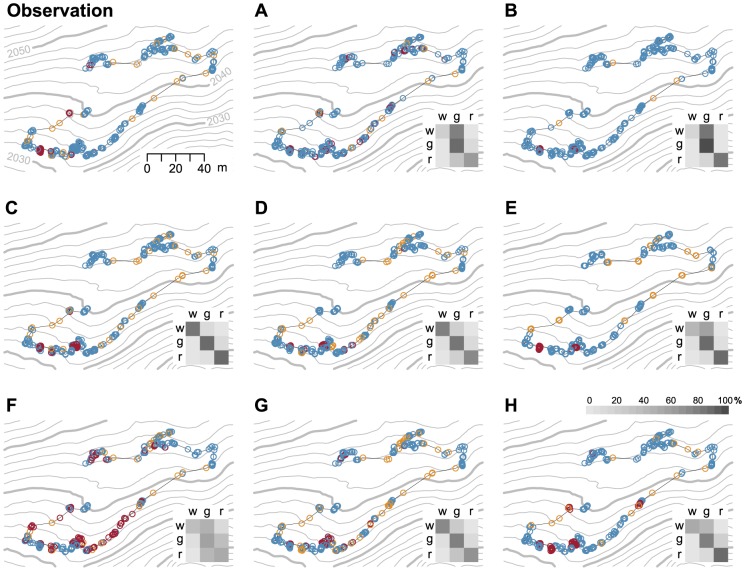
Comparison of observed and predicted behavioral states along an exemplary cow track. Colors indicate the three behavioral states of walking (gold), grazing (blue), and resting (red) as observed in the field (Observation) and as predicted by eight classification models (A - H). Gray symbols are observed additional states, which were too rare for classification. Slight jitter was added to the positions to minimize symbol overlap. The time interval between positions is 20 s, except for model E, subsampled to 60 s. Gray lines are isolines at 2 m distance (swissALTI3D, Swiss Federal Office of Topography, Wabern). The inlay shows the confusion matrix with gray-shading according to the percentage of positions in each row classified into the three behavioral states, i.e. the first row shows the percentage of positions observed as grazing and classified as walking (w), grazing (g), and resting (r).

Using the unbalanced models A and B, 76% and 81% of the positions observed as walking were classified as grazing, respectively (Figure 4). Using model C, balanced using RUS, the number of falsely classified positions was altogether balanced and was between 2% and 14%. This numbers were slightly higher and less equal in model D, balanced using SMOTE. In model E, despite data balancing with RUS, a rather high number of observed walking positions (56%) were classified as grazing. Model F produced relatively high percentages of falsely classified positions, especially observed walking and resting positions classified as grazing, and observed grazing positions classified as resting. In model G, the distribution of misclassified positions was similar to model D. In model H, 43% of observed walking was classified as grazing and 20% of grazing was classified as resting.

**Figure 5:**
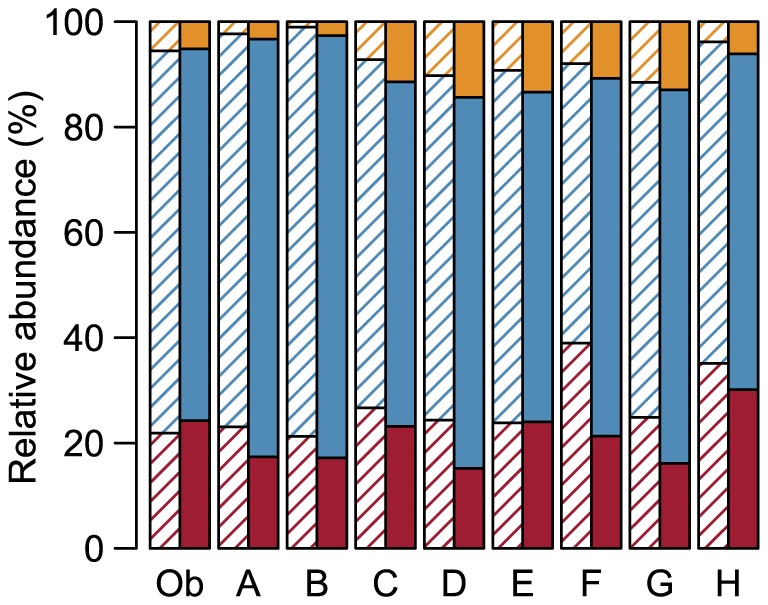
Relative abundance of the three behavioral states. Accumulated relative abundance of the states walking (gold), grazing (blue), and resting (red), as it was observed (Ob) and as it was predicted by the eight classification models (A - H) in the example track (striped columns) and in the whole data set (filled columns).

The differences between the classifications of the eight models and the visual observation were also reflected in the predicted relative abundances of the behavioral states calculated for the example track (Figure 5). Specifically, when compared to the observed reality, models A and B predicted more grazing positions. Models C to G predicted a little more walking and resting positions at the cost of grazing positions. High portions of resting positions were predicted by models F and H.

These tendencies were also present in the abundances calculated for the whole data set (Figure 5). However, most models classified fewer positions as resting and more positions as walking in the whole dataset than in the example track. Regarding the resting positions, this difference was especially large in model F.

### Importance of predictor variables for classification

**Figure 6:**
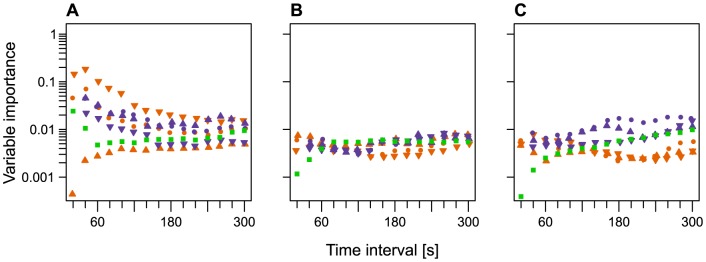
Variable importance of movement metrics in model C. Mean variable importance of movement metrics for the behavioral states walking (A), grazing (B), and resting (C) as calculated from 50 random forest realizations of model C. Colors and symbols indicate the different movement metrics cumulative distance (orange), linear distance (violet), forward distance (upward triangle), backward distance (downward triangle), mean distance (point), turning angle and mean turning angles over multiple positions (green square).

For the classification of walking, backward cumulative distances over short time intervals were most important (Figure 6a). Linear distances were less important for the state of walking than cumulative distances. Regarding the turning angles, their importance was altogether low, but the angle at a considered position, the average over three positions and the average over a large number of positions were comparably more important for the classification of walking. In contrast to walking, grazing was characterized by a large range of distance metrics and average turning angles, which is apparent from the more equal distribution of importance across time intervals (Figure 6b). This finding agrees with our field observations of the cows. What was visually observed as grazing covered quite a large range of movement speeds, i.e. almost stationary feeding when the fodder quality was good and cows were familiar with the terrain, but also dynamic grazing in order to detect the rare patches of high quality forage in the case of low food supply. The third behavior, resting, was observed in the field as a behavior of rather long duration. Similar to grazing, a high number of distance metrics was needed for correct classification, but especially mean linear distances calculated over large time intervals (Figure 6c). For the classification of resting turning angles averaged over large time intervals were more important than those averaged over small time intervals.

## Discussion

Our evaluation demonstrates that behavioral states in cows grazing heterogeneous terrain can be reliably identified with high-frequency position data alone. Such information is of great use in analyzing spatial or temporal activity patterns of grazing animals and resulting consequences for the delivery and management of ecosystem services. However, the classification of high-resolution data poses a number of challenges, which are discussed below.

### Random forest is well adapted to behavioral classification

Animal tracking data are temporally interdependent. Temporal dependence is explicitly accounted for by state-space models, which include a state-transition equation, meaning that states are modelled depending on the previous state in time [25]. In contrast, machine learning models do not consider the temporal order of the classified positions. To account for temporal dependence we used not only movement information from one recorded position to the next but across multiple positions. Hence, the models implicitly account for persistence in the behavior of the studied animal. The importance of many predictor variables for the classification of positions confirms that the use of a large range of movement metrics is justified: each behavioral state was characterized by another set of metrics.

Multiple variables calculated from the same data are often highly correlated. This multicollinearity of the predictor variables must be considered when choosing a machine learning method. Classical techniques based on eigenvectors, such as discriminant analysis, can produce inaccurate predictions if performed with collinear variables because the eigenmatrix cannot be sufficiently reduced [26], [38]. Under these circumstances, tree-based machine learning techniques such as random forest often perform better than eigenvector-based approaches [26]. Because random forest aggregates multiple random decision trees, collinear variables have less impact on results than in eigenvector-based methods. In our data set, a linear discriminant analysis conducted with the identical full variable set resulted in an overall classification accuracy which was ten percent units lower than the random forest classification.

Random forest also allows for the reduction of predictors, thereby reducing multicollinearity of predictor variables. Variables are retained based on their importance, which is calculated during the buildup of the random forest [39], [40]. Random forest models with a significantly reduced variable set yielded nearly the same accuracy as conducted with the total variable set, which confirms collinearity and an excess of predictor variables (Figure S1). However, because there was no difference in the classification result, these issues were not of concern.

A random forest model and a state-space model both including the predictor variables distance to the preceding position and turning angle did not differ significantly in classification accuracy. Adding movement metrics over multiple time steps led to a significant improvement of the random forest classification result, but to a substantial decline of accuracy in the state-space model (Figure S2). This indicates that, regarding the number of predictor variables and the ability to select the most important for classification, the random forest approach is more flexible than the state-space approach.

### Unbalanced data impairs classification accuracy of rare states

When assessing animal behavior we often deal with unbalanced data, as behavioral states are not equally frequent. While random forest classification with the unbalanced data set produced the highest overall classification accuracy of all models, only the common state of grazing was well classified, and the less frequent states of walking and resting had low classification accuracies. Such a classification with unbalanced data could be an adequate solution if only the most frequent state is of interest. However, one should note that even the null model produced a classification accuracy of 70% for grazing.

In order to avoid biased accuracies for infrequent behavioral states, our evaluations suggest conducting a balancing procedure even if the overall accuracy is higher with unbalanced than with balanced data. This is especially important if the more infrequent states are of special interest or if the frequency of states is not known *a priori*. Balancing can be done by stratified behavior observation in the field, by drawing balanced subsamples of data (e.g. by RUS), or by augmenting the less frequent states during the statistical analysis (e.g. by SMOTE). In contrast to other classification techniques, the random forest algorithm provides a convenient possibility for RUS, in which a random balanced subset is chosen for growing each individual tree of an entire forest [41]. Van Hulse et al. [42] assessed the utility of different balancing techniques in the context of different classification methods and found RUS performing well with random forest. Indeed, the same model trained with data balanced with SMOTE produced a lower overall classification accuracy and stronger differences between state-specific accuracies. In the case of state-space modelling there is no way to preselect data points prior to classification, because the original temporal order of positions is needed for modelling. Nevertheless, the state-specific accuracies of a state-space model were more balanced than those produced by a random forest model with the same predictor variables and without data balancing prior to modelling.

Data balancing also had an effect on the quantification of behavioral budgets of the tracked animals: Classification of the unbalanced data set resulted in an overestimation of the relative abundance of the most frequent state. Predicted abundances of behavioral states are also influenced by redistributions of positions between the behavioral states, which are specific for each model. A model trained with data balanced by SMOTE, for example, classified an especially high number of positions as walking, i.e. the rarest state. The linear discriminant analysis, however, resulted in a surplus of positions classified as resting. Predictions based on an accurate model will always have a higher probability of matching the real behavioral pattern of an animal than those based on a less accurate model.

### Importance of turning angle and terrain slope for behavioral classification

In contrast to our expectations, classification accuracy was not reduced by omitting turning angles from the models (Figure S3). This is surprising given the fact that turning angle is frequently a major component of state-space models of animal movement [37], [43]. Apparently, the ability of turning angle to discriminate between different behavioral states depends on the behavioral pattern of the species studied and the technical setup. In contrast to our study, possums [24], elk [32], and grey seals [44] tagged at intervals between 5 minutes and two days showed two behavioral states with quite different movement characteristics, a stationary behavior and a directed long-distance travelling behavior. The distributions of turning angle characterizing the two behavioral states were sufficiently separated, and therefore, turning angle improved classification accuracy [43]. The behavior of pasturing cows rather corresponds to a stationary, also called ‘slow-area-restricted’ [24], behavior and comprises several behavioral states with similar movement characteristics changing rapidly in time and space compared to the characteristics of stationary and travelling behavior. In our data, especially the distributions of turning angle within the behavioral states grazing and resting were weakly separated. Another possible reason for this was revealed by our test of device accuracy. Recorded positions are averages over multiple readings calculated inside the GPS device and, hence, the absolute measurement error exceeded the relative error between subsequent positions. Thus, the measurement error during resting did not result in the expected scatter plot-like recordings apparent with large turning angles.

Higher classification accuracy and a clearer separation between resting on the one hand and walking and grazing on the other hand was expected by including the environmental variable of terrain slope, as steep slopes inhibit resting, due to physical difficulty. Behavior of ranging cows is influenced by pasture terrain [45], but the inclusion of terrain slope did not help to better define their behavioral states (Figure S4). The information of terrain slope is apparently contained within the movement metrics already. Furthermore, terrain slope is a weak predictor, because it excludes resting at steep slopes but it does not exclude walking and grazing on flat pasture areas. It is weakened by the fact, that we built the state resting by combining lying on the ground and standing, of which standing is more likely on slopes than lying.

### Accurate predictions require high temporal resolution

Using a time interval of 20 s, it was possible to visually interpret the behavioral states of walking, grazing, and resting from the recorded cow tracks, which was also found by Davis et al. [46]. Postlethwaite et al. [24] reported that extended time intervals in movement models impede the identification of behavioral states. They attributed this result mainly to the fact that, as sampling time interval increased, turning angles became more uniformly distributed. We also observed that the distributions of distance metrics became more uniform with increasing sampling time interval. If we subsampled our data set at intervals of 60 s compared to 20 s, overall classification accuracy dropped by only three percent units, but walking was especially misclassified, the state which is mainly defined by large displacement at short time steps. We ensured that this effect was not due to the reduced number of positions in the data set.

### Multiple movement metrics characterize behavioral states of grazing cows

Our investigation shows that the classification of behavioral states based solely on movement characteristics between two subsequent positions is likely unsatisfactory, confirming a number of earlier, less systematic studies. Anderson et al. [20], for example, compared several distributions of mean distance between subsequent positions at time intervals from 30 s to 180 s. They showed that averaging distance over several time steps produces more distinct and narrower peaks in stationary and slow movement behavior but a flattened peak in fast movement, such as walking. This trade-off can be overcome by including more than one movement metric in the classification. Schlecht et al. [19] used four distance variables within a time interval of 3 min to classify behavioral states of grazers. In our study, the behavioral states of grazing and resting showed similar distances between subsequent positions. Hence, their distinction was particularly challenging and relied on the use of multiple movement metrics. Ungar et al. [16] pointed out that the state of walking but not resting and grazing could be distinguished using distances between subsequent positions. However, as they measured positions at 20 min and 5 min intervals, the effect of temporal resolution might also partially explain this result. Variable importance, calculated during random forest classification, clearly demonstrates the relevance of various movement metrics for the classification of behavioral states. The fact that classification based solely on measures between subsequent positions can be distinctly different from one based on multiple intervals corroborates the need to include persistence in movement models of grazing animals [37]. Two extended state-space models including movement metrics over several time steps (Figure S2) did not yet provide improved overall classification results, but more balanced state-specific accuracies.

## Conclusions

GPS data alone can be exploited for many applications. With the easy accessibility to and increased accuracy of portable, low-cost GPS receivers, inference of animal behavior from position data alone has a large potential for use in applied ecological studies. Pasture and livestock ecologists are now able to locate animal activities precisely in space and time over extended study periods. This spatio-temporal information improves the analysis and understanding of vegetation patterns and biogeochemical processes, and contributes to the development of sustainable land use strategies.

In this study, we have advanced behavioral classification from GPS data alone by evaluating a wide range of approaches involving movement metrics at multiple time intervals. We found random forest classification models very helpful in an applied context of behavior estimation, especially in their ability to handle many collinear predictor variables and highly unbalanced class frequencies. Despite the fact that there is no underlying process model, calculating variable importances facilitates the interpretation of results. They showed that a high temporal resolution in the collection of position data and the use of multiple distances at a range of time steps were prerequisites for a satisfactory classification. This supports further efforts in development of movement models that consider persistence and memory in the behavior of grazing animals.

Our investigation also demonstrated that the fit of such complex classification models would not have been possible without direct field observation. Comparisons with other studies show that classification accuracy and the importance of predictor variables is highly context-dependent. Hence, direct field observations of animals in the environment under study are indispensable for understanding their behavioral patterns and their expressions in different environmental and social constellations.

## Supporting Information

Figure S1 **Comparison of random forest models with full and reduced set of predictor variables.** Full models (circles) were reduced (triangles) using package varSelRF [39], which iteratively excludes predictor variables with low importance until the classification accuracy drops. Shown is the percentage of correctly classified states in joint sequences together with 50% and 95% quantile intervals for individual sequences, overall (black) and for the three behavioral states walking (gold), grazing (blue) and resting (red). For specifications of models see Table 1.
(EPS)

Figure S2
**Extensions to the state-space model.** Percentage of correctly classified states in joint sequences together with 50% and 95% quantile intervals for individual sequences, overall (black) and for the three behavioral states walking (gold), grazing (blue) and resting (red). Model H was a state-space model in which the likelihood of being in a state and the switching probability depended on the position at time t-1 and the turning angle. Model H1 was similar to H, but the likelihood of being in a state depended on 30 distance metrics (distances to positions at other time intervals than t-1) and turning angle. Model H2 was a state-space model in which also the switching probability depended on 30 distance metrics and turning angle, i.e. 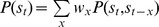, where s is the state, x is the time step of the included movement metrics and w are weights for each of the time steps of the included movement metrics. Variable importances calculated from model C were used to identify the distance metrics to be included. The ten distance metrics with the highest variable importance for each state were included and their distributions modeled by independent Weibull distributions.(EPS)

Figure S3 **Comparison of random forest models including and excluding turning angle.** Circles represent models with the respective distance and turning angle metrics, triangles represent models with distance metrics only. Shown is the percentage of correctly classified states in joint sequences together with 50% and 95% quantile intervals for individual sequences, overall (black) and for the three behavioral states walking (gold), grazing (blue) and resting (red). For specifications of models see Table 1.
(EPS)

Figure S4 **Random forest classification including terrain slope.** Percentage of correctly classified states in joint sequences together with 50% and 95% quantile intervals for individual sequences, overall (black) and for the three behavioral states walking (gold), grazing (blue) and resting (red). Model C was a random forest model with all movement metrics. Model C1 was similar to Model C but included terrain slope. Terrain slope at each GPS position was extracted from altitudinal data with a resolution of 2 m (swissALTI3D, Swiss Federal Office of Topography, Wabern). The absolute accuracy of all three dimensions in this data was 0.5 m for data below 2000 m asl and 1–3 m for data above 2000 m asl.
(EPS)

Dataset S1 **Original GPS tracking data with observed cow behavior.**
(TXT)
